# Comparison of cancer subtype identification methods combined with feature selection methods in omics data analysis

**DOI:** 10.1186/s13040-023-00334-0

**Published:** 2023-07-07

**Authors:** JiYoon Park, Jae Won Lee, Mira Park

**Affiliations:** 1 Department of Statistics, Korea University, 145 Anam-Ro, Seongbuk-Gu, Seoul, 02841 South Korea; 2 Department of Preventive Medicine, Eulji University, 77 Gyeryong-Ro, Jung-Gu, Daejeon, 34824 South Korea

**Keywords:** Cancer subtype identification, Clustering, Feature selection, Omics data

## Abstract

**Background:**

Cancer subtype identification is important for the early diagnosis of cancer and the provision of adequate treatment. Prior to identifying the subtype of cancer in a patient, feature selection is also crucial for reducing the dimensionality of the data by detecting genes that contain important information about the cancer subtype. Numerous cancer subtyping methods have been developed, and their performance has been compared. However, combinations of feature selection and subtype identification methods have rarely been considered. This study aimed to identify the best combination of variable selection and subtype identification methods in single omics data analysis.

**Results:**

Combinations of six filter-based methods and six unsupervised subtype identification methods were investigated using The Cancer Genome Atlas (TCGA) datasets for four cancers. The number of features selected varied, and several evaluation metrics were used. Although no single combination was found to have a distinctively good performance, Consensus Clustering (CC) and Neighborhood-Based Multi-omics Clustering (NEMO) used with variance-based feature selection had a tendency to show lower *p*-values, and nonnegative matrix factorization (NMF) stably showed good performance in many cases unless the Dip test was used for feature selection. In terms of accuracy, the combination of NMF and similarity network fusion (SNF) with Monte Carlo Feature Selection (MCFS) and Minimum-Redundancy Maximum Relevance (mRMR) showed good overall performance. NMF always showed among the worst performances without feature selection in all datasets, but performed much better when used with various feature selection methods. iClusterBayes (ICB) had decent performance when used without feature selection.

**Conclusions:**

Rather than a single method clearly emerging as optimal, the best methodology was different depending on the data used, the number of features selected, and the evaluation method. A guideline for choosing the best combination method under various situations is provided.

## Introduction

In the era of precision medicine, cancer subtype identification, which aims to divide patients into subgroups with distinct clinical phenotypes such as survival time, is of the utmost importance. Since cancer is a heterogeneous disease, cancer types can have several subtypes with different phenotypic and molecular profiles [1]. The classification of patients is essential for the early diagnosis of cancer and the provision of adequate treatment. An early diagnosis of cancer can increase patients’ survival probability, and the identification of a clinically relevant subtype is crucial for selecting and administering the most effective treatment, as different cancer subtypes may respond differently to specific treatments [2–5]. With the advancement of large-scale omics technologies, cancer subtypes have been identified in multiple cancers using mRNA and microRNA expression levels, methylation data, and multi-omics data [6–9].

Numerous cancer subtyping methods have been developed, and these methods can be divided into two types: supervised and unsupervised learning. In supervised learning, classification takes the true class into account and classifies a new patient to the correct label, whereas clustering based on unsupervised learning does not use class labels when patients are grouped into similar types. One of the major limitations of classification analysis is that it cannot identify novel subtypes. The current subtypes of breast cancer were proven to be highly ambiguous, resulting in an inaccurate classification of new patients [10]. In this paper, we focus on the unsupervised approach instead of the classification approach. Among unsupervised methods, Consensus Clustering (CC) [11] is the state-of-the-art method for cancer subtyping that uses single-omics biological data to compute patient similarity. Nonnegative Matrix Factorization (NMF) has also been applied in single-omics cancer datasets [12]. More recent studies have utilized multiple datasets and focused more on the integration of these datasets, as technological advances have made such multi-view analyses possible. For instance, the proper integration of genome, transcriptome, and epigenome information would enhance the predictability of subtyping, if such a wealth of data is available for a set of samples. These integrative clustering methods include iCluster [13], iClusterPlus [14] and iClusterBayes (ICB) [15], which are based on joint statistical modeling and depend on the adequacy of the statistical assumptions. Similarity network fusion (SNF) [16] constructs a fused network using a patient similarity network constructed from each data type. Neighborhood-Based Multi-Omics Clustering (NEMO) [17] and Perturbation Clustering for Data Integration and Disease Subtyping (PINS) [18] are also similarity-based approaches.

An important procedure when identifying cancer subtypes is feature selection for detecting genes that contain important information about the cancer subtype [19, 20]. Feature selection is often used to reduce the dimensionality of high-dimensional data, thus dramatically reducing the time taken to run the algorithms. Informative genes should be selected prior to patient clustering, as it is presumed that the expression of only a subset of genes is affected by the subtype, and the inclusion of irrelevant genes can disturb proper clustering. Thus, the choice of the feature selection method is equally important in subtype identification as the choice of the clustering method. Feature selection without labels should utilize the characteristics of the genes across samples. Recent efforts to identify useful feature selection methods in genomic setting have shown the importance of selecting informative genes in cancer subtyping [21–23].

There are various ways to classify feature selection algorithms. One approach is to consider this issue from the perspective of data, including statistical measure-based, probability measure-based, similarity measure-based, sparse learning-based, and evolutionary algorithm-based feature selection methods [24, 25]. However, feature selection methods are most commonly classified as filter, wrapper, and embedded methods [24, 26, 27]. In filter methods, a learning algorithm is not used to evaluate a subset of features; instead, features are filtered based on a criterion called a measure of feature relevance. The measures include variance, correlation, the F-statistic, mutual information, and information gain [24, 25]. In recent research, Maximum Clique and Edge Centrality (MCEC) [28], and Dual Regularized Unsupervised Feature Selection based on Matrix Factorization and Minimum Redundancy (DR-FS-MFMR) [29] have been proposed. MCEC utilizes social network analysis to select a subset of genes that meets the Minimum-Redundancy Maximum Relevance (mRMR) criterion, and it has the advantage of being able to determine the optimal number of geneset automatically [28]. DF-FS-MFMR obtained the optimal feature set using matrix factorization and correlation information. The objective function is solved using an optimization algorithm and its convergence analysis [29]. Filter methods are computationally efficient and independent of the classification or clustering algorithms. Since the main purpose of cancer subtype identification is to explanatorily find unknown classes in a large dataset, we focus on filter model. Specifically, the variance (VAR), median (MED), median absolute deviance (MAD), Dip test (DIP), mRMR, and MCFS [22, 30–32] methods are considered. These methods are discussed further in "Feature selection methods" section.

Wrapper methods use specific learning algorithms to evaluate the performance of a feature subset [33]. Forward and/or backward procedures to find the optimal subset are often used. For example, Sequential Forward Floating Selection (SFFS) starts with an empty feature set and iteratively adds the best feature that improves the model performance [34]. Recursive Feature Addition (RFA) adds features to the model one at a time, starting with the most important feature, and continuing until the desired number of features is reached [35]. Guided Regularized Random Forest (GRRF) trains a random forest model on the entire feature set and uses feature importance scores to guide a regularization algorithm that selects the most important features [36]. The wrapper method has the advantage of considering the interaction between variables, but has the disadvantage of high computational cost.

Embedded methods differ from wrapper methods in that the optimal feature subset is built into the classifier construction. PSO-GWO is an example, which is a multi-objective feature selection method using Newton's law-based Particle Swarm with Grey Wolf optimization to minimize the classification error rate while performing feature selection [25]. Deep Feature Selection is another embedded method. It trains a deep neural network to perform feature selection and classification simultaneously by using the hierarchical representation of the input features and selecting the most informative features at each layer [37]. Lastly, two-layer feature selection methods have been proposed, including the Genetic Algorithm and Elastic Net. Elastic Net combines L1 and L2 regularization to select features that are both sparse and correlated, hence encouraging both sparsity and correlation among the selected features [38]. Embedded methods are capable of better handling high-dimensional data, and less prone to overfitting. They are also able to simultaneously optimize and select features, making a separate feature selection step unnecessary. Embedded methods reduce the computational time required to reclassify compared to wrapper methods, although they are still more time-consuming than filter methods [39]. Numerous feature selection methods have been proposed, each of which has advantages and drawbacks.

Several attempts have been made to review and compare existing unsupervised clustering methods for subtype identification [40–43]. A systematic comparison of nine multi-omics clustering algorithms, including PINS [18], SNF [16] and ICB [15], was conducted using 10 The Cancer Genome Atlas (TCGA) datasets [40]. More recently, 13 unsupervised integrative methods were benchmarked on eight simulation datasets [41], but neither study compared the accuracy of the algorithms using a real cancer dataset. In another study, two Bayesian approaches and four matrix factorization approaches were compared using simulated data and the TCGA breast cancer dataset [42]. Moreover, five network-based methods and three statistic-based integration methods, as well as PINS [18] and Subtype-GAN [44], which do not fall into either of those categories, were reviewed using simulated data and the TCGA breast cancer dataset [43]. However, neither of those studies took feature selection into account prior to clustering. Most review papers that used TCGA data focused more on the integrative aspect of the methodologies, and again, the combinations of subtype identification and feature selection methods were not considered [40].

In this study, we compared the performance of combinations of feature selection and clustering methods, evaluated on four TCGA datasets of cancers with different characteristics. We considered six methods of feature selection and six clustering methods for subtype identification. The clustering methods are either state-of-the-art or commonly used in subtype identification, and only those available in the R programming language were chosen. Thus, a total of 24 combinations were compared in two mRNA gene expression datasets without labels, and 36 combinations were compared in two mRNA gene expression datasets with gold-standard labels using different settings. Furthermore, for each feature selection method, we compared the results of two different numbers of selected features along with the results when all features were used.

In "Materials and methods" section, we briefly review several current methods for cancer subtyping and feature selection. The simulation scheme and materials are also described. Comparative results of the methods’ performance are shown in "Results" section, and a short discussion is presented in "Discussion" section.

## Materials and methods

The usual workflow for identifying cancer subtypes is as follows. After data preprocessing, which includes missing value imputation and normalization, we select informative genes using a feature selection method to solve the redundancy problem. A clustering method for subtype identification is then applied. The result is validated by checking the silhouette score or log-rank test. We briefly review the selected feature selection methods, clustering methods, and evaluation methods as follows.

### Feature selection methods

We considered six feature selection methods based on four criteria. First, we selected genes with high expression levels. We computed the median expression level (MED) for each gene across samples and selected the ones with high median values. The second criterion was based on variation. Genes with a large variability of expression are expected to contain variations caused by the subtype of cancer [45–47]. We selected the genes with large variability using two measures: variance (VAR) and the median absolute deviance (MAD). The third criterion was based on modality, which refers to whether the distribution of gene expression levels has two or more peaks (modes). The peaks of the distribution are thought to represent the different subtypes of a disease, and informative genes can thus be detected by checking the multimodality of the gene expression distribution. There exist several ways of checking the bimodality of genes. These methods include parametric tests such as the Bimodality Index (BI) [48] and nonparametric tests such as the Variance Reduction Score (VRS) [49]. In this study, we used DIP, which extends the bimodality problem to the multimodality problem. DIP computes the maximum difference between the empirical distribution function and the unimodal distribution that minimizes the maximum difference. Genes with low *p*-values are selected. The fourth criterion was based on the relevance of informative genes and their target variables for the datasets with true class labels. We selected genes with a high information criterion using two measures: mRMR [30] and Monte Carlo Feature Selection (MCFS) [31]. mRMR aims to identify a subset of genes that are most relevant to a class while minimizing redundancy among selected features. Relevance can be calculated by using the F-statistic or Mutual Information (MI), and redundancy can be calculated by using Pearson correlation coefficients or MI [30]. MCFS is a computer-intensive method relying on Monte Carlo approach. MCFS identifies relevant features by randomly selecting a subset of genes and evaluating their relevance using decision trees or support vector machines. This process is repeated multiple times and the most frequently selected feature is finally selected as the most relevant feature. It should be noted that mRMR and MCFS are not always applicable as they require labeled data for selection, which may not always be available or feasible.

### Subtype identification methods

We considered six popular subtype identification methods: CC, NMF, PINS, ICB, SNF, and NEMO. In all methods, the goal is to group the samples into $k$ clusters, given a dataset *D* of *m* genes and *n* samples.

#### Consensus Clustering (CC)

CC is a model-independent resampling-based method for single genomic datasets. CC achieves consensus across multiple clustering runs. It also involves determining the number of clusters and assessing the stability of the clusters [11]. The algorithm consists of two steps: resampling and clustering. In the resampling step, $H$ perturbed datasets ${D}^{\left(1\right)},{D}^{\left(2\right)},\dots ,{D}^{\left(H\right)}$ are generated from the original dataset using a pre-specified resampling algorithm without replacement. In genomic datasets, gene resampling can also be used, in which the candidate genes can be given different weights if prior information is available. The goal of the clustering step is to partition a given dataset $D$ into a set of $k$ clusters. In the clustering step, a connectivity matrix ${M}^{\left(h\right)}$ and an indicator matrix ${I}^{\left(h\right)}$, both of size $n\times n$, are created from each of the perturbed datasets ${D}^{\left(h\right)}$ using the pre-specified clustering algorithm for $h=1,\dots ,H$. Using the connectivity and indicator matrices, the consensus matrix $\mathcal{M}$ is generated, which represents the consensus of the connectivity matrices. The ($i,j$)^th^ element of $\mathcal{M}$ is obtained by

1 $$\mathcal{M}\left(i,j\right)=\frac{\sum_{h}{M}^{\left(h\right)}\left(i,j\right)}{\sum_{h}{I}^{\left(h\right)}\left(i,j\right)}$$

where ${M}^{\left(h\right)}\left(i,j\right)$ is an indicator of whether items *i* and *j* belong to the same cluster and ${I}^{\left(h\right)}\left(i,j\right)$ is indicator of whether both items *i* and *j* are present in $D^{\left(h\right)}$.

To determine the number of clusters $k$, an ad-hoc technique can be adopted. A perfect consensus results in all the elements of $\mathcal{M}$ having values of either 0 or 1. Thus, we can select the optimal $k$ corresponding to the cleanest consensus matrix ${\mathcal{M}}^{(k)}$, where the distribution of elements is skewed toward 0 or 1. A histogram of the consensus matrix elements and the resulting empirical cumulative distribution (CDF) can be used to find the best $k$, where the shape of the empirical CDF of the true $k$ would approach the ideal step function. The CC method can be implemented using the R package *ConsensusClusterPlus*.

#### Nonnegative Matrix Factorization (NMF)

The goal of NMF is to find a small number of metagenes from thousands of genes based on decomposition by parts [12]. Each metagene is defined as a positive linear combination of $m$ genes, and one can approximate the gene pattern of samples as a positive linear combination of metagenes. Then, the NMF algorithm clusters patients with regard to each of the metagenes.

To obtain $k$ metagenes, an $m\times n$ data matrix $D$ is decomposed into two nonnegative matrices ${U}_{m\times k}$ and ${V}_{k\times n}$ (i.e., $D\sim UV$). The element ${u}_{ij}$ of matrix $U$ represents the coefficient of the ${i}^{th}$ gene in the ${j}^{th}$ metagene, and the element ${v}_{ij}$ of matrix $V$ represents the expression level of the ${i}^{th}$ metagene of the ${j}^{th}$ sample. With the random initialization of $U$ and $V$, the algorithm iteratively updates the two matrices. Then, $V$ is used to group $n$ samples into $k$ clusters, where each sample is placed into a cluster in which it shows the highest expression of the metagene in matrix $V$. Though NMF does not assume a hierarchical structure of clusters, it shows a tendency to discover substructures of existing clusters as the number of clusters increases [12].

The NMF method builds on the CC algorithm to evaluate clusters quantitatively. NMF evaluates the robustness of decomposition quantitatively by assessing how much clusters vary in each run. Using the same concept of CC, the connectivity matrix and the resulting consensus matrix are obtained. The off-diagonal elements of consensus matrix represent the probability that a pair of samples belongs to the same cluster. The reordering of a consensus matrix using the average linkage hierarchical clustering provides a visual inspection of clustering stability. Furthermore, the quantitative measure of clustering robustness can be obtained as the cophenetic correlation coefficient of rank $k$ [50].

This method can be implemented using the *NMF* package in R. The *NMF* package provides several different NMF algorithms, published by different authors.

#### Similarity Network Fusion (SNF)

SNF is a non-Bayesian network-based method for integrating and finding cancer subtypes [51]. In the first step, SNF constructs a similarity network between patients for each datatype. A similarity network is represented as a graph ${\varvec{G}}=({\varvec{V}},{\varvec{E}})$, where the vertices ${\varvec{V}}$ represent the patients $\{{d}_{1},\dots ,$ ${d}_{n}\}$ and the edges ${\varvec{E}}$ represent the degree of similarity between patients [16]. The elements of the similarity matrix are the weights of the edges between patients calculated using the scaled exponential similarity kernel. In the second step, the network fusion step, the similarity networks are iteratively updated using nonlinear combinations so that they become more and more alike, converging to the final fused network.

Through this process, weak similarities of low-weight edges are considered as noise and disappear, while only strong similarities of high-weight edges remain. The SNF algorithm uses full and sparse kernels to compute the fused matrix. The full kernel matrix carries the full information about similarity to all others, while the sparse kernel matrix encodes the similarity using the $k$-nearest neighbors for each patient. The number of neighbors is set to be the ratio of the number of samples to the number of clusters if it is known, but if the number of clusters is not known, the authors recommended using 6, which is a crude estimate of the number of clusters observed in cancer datasets [16, 17]. Finally, given the fused graph, patients are clustered using spectral clustering, which is known to be effective in capturing the global structure of a graph [52].

SNF is generally used for integrating multi-omics data, but can also be used for clustering in single omics setting. This method can be implemented using the package *SNFtool* in R.

#### Perturbation clustering for data integration and disease subtyping (PINS)

The PINS algorithm, similar to the CC algorithm, uses the resampling and clustering technique to discover cancer subtypes. As it assumes that the true subtypes are stable with regards to small changes in features, new datasets are first obtained by repeatedly perturbing the data $H$ times, and the samples from the resulting datasets are then partitioned using the pre-specified clustering algorithm. The goal of the PINS algorithm is to identify the partitioning that is least affected by perturbation with regard to the number of clusters [18].

Perturbed datasets can be generated by adding Gaussian noise. PINS sets the variance of the perturbation noise equal to the median variance of the original data. The clustering stability is evaluated by comparing the partitions obtained from the original data to those obtained from the perturbed datasets. Using the concept of CC, the original connectivity matrix is obtained from the original data, and the perturbed connectivity matrix is obtained by calculating the average of the perturbed trials. The perturbed connectivity matrix will always reflect the true structure of the data, since PINS assumes that for truly distinct subtypes, the true connectivity between samples is recovered when the data are perturbed regardless of the number of clusters $k$. The difference matrix is then calculated as the absolute difference between the original and the perturbed connectivity matrices. The best number of clusters is the one that minimizes this difference [41].

For each value of $k$, the empirical CDF of the difference matrix and its area under the curve (AUC) is obtained, and the optimal value of $k$ is selected to be the one with the highest AUC. When used in R, the perturbation is repeated 200 times and the partition samples are clustered using hierarchical clustering. This method can be implemented using the package *PINSPlus*.

#### iClusterBayes (ICB)

IClusterBayes is a Bayesian latent variable model that can jointly model omics data of continuous and discrete types [15]. The integrative clustering algorithm, iCluster, reduces the dimensionality of data for clustering and integrates various data types [13]. The iCluster algorithm seeks a pattern that is consistent among multiple data types and patterns that are unique in individual data types by separating the covariance between data types and the variance within a data type. The method incorporates joint latent variable modeling in calculating the principal components, thereby estimating the latent tumor subtype that can account for all data types.

ICB is known to overcome the limitations of iCluster in terms of statistical inference and computational speed. It adds an extra penalty term such as LASSO for the purpose of feature selection [53]. In single omics data, it is similar to principal component analysis, where the first few principal components that capture most variation in the data are used to cluster the samples. The high-dimensional space is projected to a low-dimensional subspace, where each sample is associated with a latent variable ${{\varvec{z}}}_{{\varvec{i}}}=\left({z}_{i1},\dots ,{z}_{i(k-1)}\right), i=1,\dots ,n$ that follows a standard multivariate normal distribution $MVN\left(0,\boldsymbol{ }{{\varvec{I}}}_{\left({\varvec{k}}-1\right)}\right)$ [15]. Through joint modeling, ${{\varvec{z}}}_{{\varvec{i}}}$ can be used not only to capture the major variations of the data, but also to distinguish the driver features for clustering.

By applying $k$-means clustering to mean ${{\varvec{z}}}_{{\varvec{i}}}$ values, the samples are clustered into $k$ subtypes in the latent variable space. IClusterBayes requires users to select the optimal number of clusters by comparing the Bayesian information criterion or deviance ratio for each $k$. This method can be implemented using the R package *iClusterPlus*.

#### Neighborhood-based multi-omics clustering (NEMO)

In real data, some patients have measurements for only a subset of omics. NEMO is an algorithm specialized in the clustering of these partial multi-omics datasets without having to impute missing data [17]. In the first step, NEMO builds on similarity-based multi-omics methods, such as SNF, to construct the patient similarity matrix. Then, NEMO modifies the similarity matrix to a relative similarity matrix based on radial basis function kernel. For omics $l,$ the relative similarity $R{S}_{I}\left(i,j\right)$ is defined as the similarity between sample *i* and *j* to *i*’s *k* nearest neighbors relative and to *j*’s *k* nearest neighbors.

2 $$R{S}_{l}\left(i,j\right)=\frac{{S}_{l}(i,j)}{{\sum }_{r\in {\eta }_{li}}{S}_{l}(i,r)}I\left(j\in {\eta }_{li}\right)+\frac{{S}_{l}(i,j)}{{\sum }_{r\in {\eta }_{lj}}{S}_{l}(r,j)}I\left(i\in {\eta }_{lj}\right)$$

where ${S}_{l}(i,j)$ is the $\left(i,j\right)^{\mathrm{th}}$ element of the similarity matrix and ${\eta }_{lj}$ refers to the k nearest neighbors within omics $l$ [17]. Then, by averaging the relative similarity in the different similarity networks for each pair of samples, it enables the analysis of partial data. In the final step, samples are clustered to identify subtypes’ spectral clustering for average relative similarity.

The number of clusters is selected using the modified eigengap method [52]. The number of neighbors is also selected in the same manner as SNF. The R code for NEMO can be downloaded from the *github* repository: https://github.com/Shamir-Lab/NEMO or implemented using the R package *NEMO*. NEMO requires pre-installation of the R library *SNFtool* and uses parts of its code*.*

### Performance metrics

To assess the performance of methods, we used several performance metrics: *p*-values from the log-rank test, the silhouette score, the Adjusted Rand Index (ARI), and Normalized Mutual Information (NMI). Computational complexity is also considered.

First, the log-rank test is used to check the significance of differences in the survival profiles between the obtained clusters. The log-rank test assumes that the clusters of patients are different in a biologically meaningful way if the difference between their survival distributions is significant. The silhouette score was also used to check clustering robustness. The silhouette score is also used to measure compactness within clusters and separation across subtypes. It is often used as a measure of clustering in unsupervised learning [16, 18]. For each observation *i*, the silhouette for patient *i* is defined as

3 $$S(i)=(b(i)-a(i))/(max(a(i),b(i)),$$

where *a(i)* is the average distance between each point within the same cluster and *b(i)* is the lowest average distance to all other patients in different clusters [16]. We used the mean value of silhouettes for all the observations, and called it the silhouette score. Its value ranges from -1 to 1, and a high value indicates that the object is well matched to its own cluster.

For datasets with true class labels, the performance was evaluated using additional measures of accuracy. The performance accuracy in the datasets was assessed by calculating ARI and NMI. Since both measures are normalized, it is possible to compare them between different clustering methods with different numbers of clusters [54, 55]. ARI assesses cluster validation by measuring the agreement between two classification results, one of which is defined by external criteria. For two partitions C and C’ in a set of *S* of *n* elements, let ${n}_{11}$ be the number of pairs of elements in *S* that belong to the same cluster in both C and C’, ${n}_{00}$ be the number of pairs that are in different subsets in C and in C’, ${n}_{10}$ be the number of pairs that are in the same cluster in C but in different clusters in C’, and ${n}_{01}$ be the number of pairs that are indifferent clusters in C but belong to the same cluster in C’. The Rand index (RI) is a way to compare the similarity of results between two clustering methods and is defined as shown by Santos and Embrecht [54].

4 $$RI=\frac{n_{11}+n_{00}}{n_{11}+n_{00}+n_{10}+n_{01}}=\frac{n_{11}+n_{00}}{{}_n^{}C_2}.$$

ARI is a correction of RI taking into account that some agreement between the two clusters may occur by chance, and defined as

5 $$ARI=\frac{RI-E(RI)}{\mathrm{max}\left(RI\right)-E(RI)}$$

where E(RI) and max(RI) are the expected and maximum values of RI, respectively [56]. The higher the ARI value, the closer the two clusters are to each other. It ranges from -1 to 1, where 1 indicates perfect agreement, 0 indicates random agreement, and -1 indicates that the two clusters are completely different.

NMI is a normalization of the MI score to scale the results between 0 and 1. 0 means no MI, while 1 means perfect correlation. MI accounts to the amount of information that can be extracted from a distribution regarding a second one. NMI is defined as

6 $$\mathrm{NMI}(\mathrm{Y},\mathrm{C})=(2\times \mathrm{I}(\mathrm{Y};\mathrm{C}))/(\mathrm{H}(\mathrm{Y})+\mathrm{H}(\mathrm{C}))$$

where Y and C are true labels and clusters, respectively, and H(.) and I(.;.) represent entropy and MI, respectively [56, 57].

Computational efficiency was also measured by calculating the total running time taken for each algorithm for all data types, with and without class labels.

### Comparisons

Combining the six feature selection methods and six subtype identification methods introduced above, the optimal combination of methods for clustering cancer patients into appropriate groups was considered. Thus, a total of 24 combinations of feature selection and clustering methods were taken into account in two datasets without true class labels, and a total of 36 combinations were taken into account for two datasets with gold-standard labels. For a fair comparison, we set the number of variables selected in all methods to be the same. Either 500 or 2000 informative genes were selected when feature selection was conducted, and we compared these results with those obtained when all genes are used. The datasets used are the preprocessed mRNA expression datasets of four different cancer types of TCGA benchmark analysis downloaded from http://acgt.cs.tau.ac.il/multi_omic_benchmark/download.html [40]. Among the 10 preprocessed TCGA datasets, four datasets of various dimensions and data types were used in this paper: acute myeloid leukemia (AML), glioblastoma multiforme (GBM), breast invasive carcinoma (BIC), and colon adenocarcinoma (COAD). The AML dataset comprises 173 samples and 19,940 genes, GBM 528 samples and 12,042 genes, BIC 671 samples and 20,249 genes, and COAD 260 samples and 17,261 genes. The data type is RNA-seqv2 level 3 RSEM genes normalized for AML, HT-HG-U133A microarray data for GBM, and HTSeq. FPKM level 3 for BIC and COAD. The TCGA datasets used for comparison can be divided into two categories: two datasets evaluated using true class labels and two datasets evaluated without using true class labels. The former category had an additional measure of assessment, as the presence of true class labels allowed classification accuracy to be measured. A problem with subtype identification using TCGA datasets is that there is no gold standard for these cancer datasets, but a previous study [58] has been done by the TCGA group in an effort to identify the subtypes for BIC and COAD patients, and these subtypes were considered as true labels in this paper in order to evaluate the performance of the methods, as done in previous TCGA benchmark studies [21, 43].

**Fig. 1:**
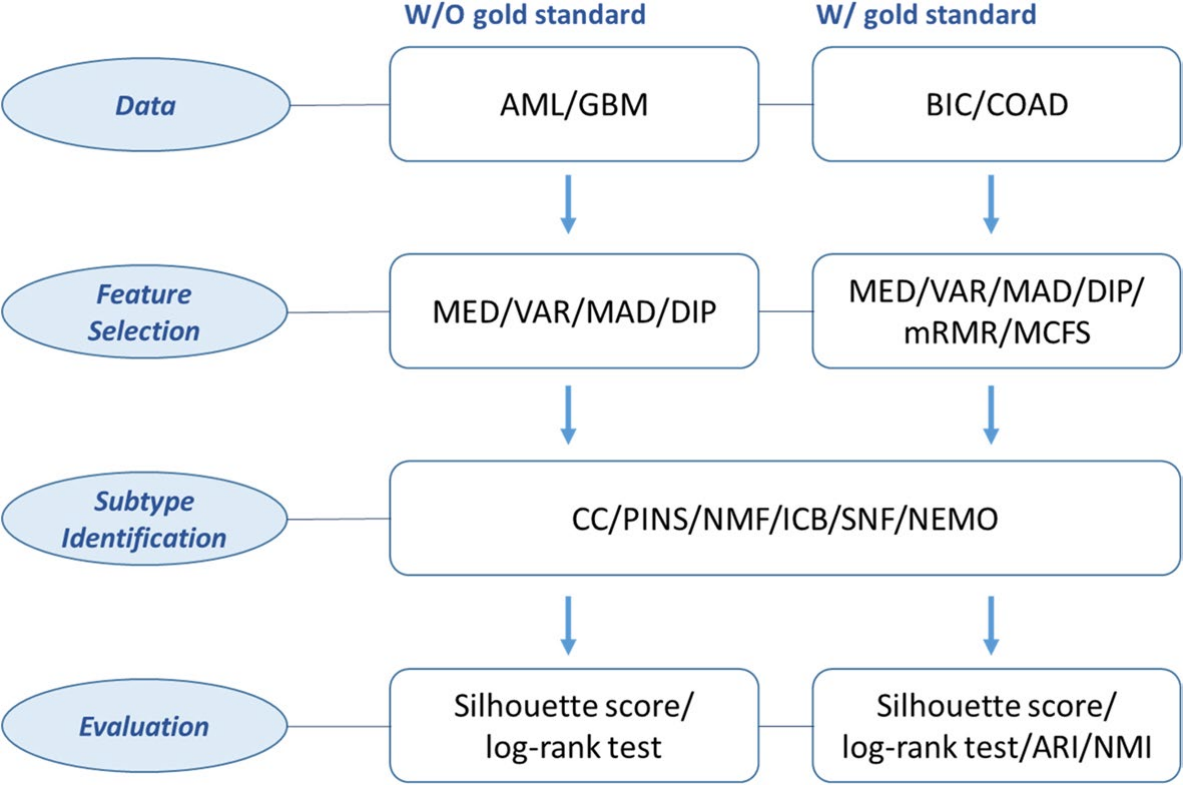
Flowchart of the comparison procedure

The datasets were preprocessed as follows: patients and features with more than 20% missing values were removed, and missing values were imputed using $k$ nearest neighbor imputation. The datasets were log-transformed for all clustering methods, and were normalized for CC, SNF, and NEMO methods. Four or six feature selections and six subtype identification methods were used for comparison. A detailed scheme of the comparisons is presented in Table 1, and a flowchart is shown in Fig. 1.

**Table 1:** Scheme for the comparisons

		**Methods**	**Software**	**Ref**	
**Feature selection**		MED	R	[21]	
		VAR	R	[21]	
		MAD	R	[21]	
		DIP	R	[32]	
		mRMR	R	[47]	
		MCFS	R	[48]	
**Number of selected genes**		ALL			
		500			
		2000			
**Subtype Identification**	Resampling-based	CC	R		[11]
		PINS	R		[18]
	Dimension reduction	NMF	R, Matlab		[12]
	Statistical model	ICB	R		[15]
	Similarity-based	SNF	R, Matlab		[16]
		NEMO	R, Matlab		[17]
**Evaluation**	All datasets	Silhouette width			
		Log-rank test Computation time			
	With gold standard only	ARI, NMI			
**Datasets**	Without gold standard	AML			
		GBM			
	With gold standard	BIC			
		COAD

The suggested number of clusters $k$ was used for the subtype identification methods, except for CC and ICB, which requires the user to specify $k$. The maximum number of clusters was set to be 10 for all methods. The criteria for determining the $k$ value in the proposed approach are shown in Table 2.

**Table 2:** Criteria used for determining the number of clusters

Subtype Identification Methods	Determination of the number of clusters k
CC	Choose k in an adhoc way that produces a consensus matrix that corresponds to the cleanest consensus matrix, i.e. a matrix with all entries with either 1 or 0
NMF	Choose k where the magnitude of the cophenetic correlation coefficient which indicates the dispersion of the consensus matrix begins to fall significantly
SNF	Chooses *k* by using spectral clustering that aims to minimize ratiocut
PINS	Chooses *k* that minimizes the absolute difference between the original connectivity matrix and the perturbed connectivity matrices
ICB	Choose *k* in an ad hoc way by selecting *k* where the Bayesian Information Criterion (BIC) value reaches the minimum or where the deviance ratio reaches a plateau which both indicate that the model fits the data best when the samples are divided into *k* + *1* subtypes. We used both BIC and deviance ratio to select *k*
NEMO	Chooses *k* using the eigengap method by selecting *k* that maximizes the product of *k* and the difference between the eigenvalues of the average relative similarity matrix of *k* + *1* and *k*

## Results

### Subtyping without feature selection

Table 3 shows the subtyping results for the four cancer datasets without feature selection. The number of clusters varied among the clustering methods and datasets, but NEMO and ICB showed a tendency to generate larger numbers of clusters than other methods. NMF showed the best performance in the AML and GBM datasets in terms of the silhouette score, whereas the methods showed low scores overall in the BIC and COAD datasets. In terms of the *p*-value for the log-rank test, CC consistently showed decent performance in clustering patients with different survival distributions. ICB had the lowest *p*-value in the GBM and BIC datasets. NEMO was the only clustering algorithm that showed a significant *p*-value under the 5% significance level in the COAD dataset. In terms of computational efficiency, NMF showed an overwhelmingly long running time, and ICB showed the second longest running time. On the contrary, SNF and NEMO took the shortest time to run.

**Table 3:** Subtyping results for each cancer dataset without feature selection

Dataset		Subtyping Identification Methods					
		CC	PINS	NMF	ICB	SNF	NEMO
AML	No. of clusters	5	5	2	5	7	7
	sil. score*	0.02	0.02	0.13	0.05	0.02	0.04
	*p*-value**	0.01	0.01	0.10	0.06	4.0E-03	0.02
GBM	No. of clusters	3	3	2	4	2	6
	sil. score	0.11	0.15	0.16	0.13	0.14	0.10
	*p*-value	0.01	0.39	0.46	0.01	0.28	0.03
BIC	No. of clusters	3	3	2	4	4	4
	sil. score	-0.01	0.06	0.03	0.00	0.02	0.02
	*p*-value	0.05	0.37	0.09	0.03	0.70	0.41
COAD	No. of clusters	4	3	2	4	3	3
	sil. score	0.06	0.08	0.03	0.02	0.08	0.08
	*p*-value	0.53	0.10	0.26	0.23	0.15	0.04

^*^sil. score: silhouette score; **p-value: *p*-value for the log-rank test

### Log-rank test

**Fig. 2:**
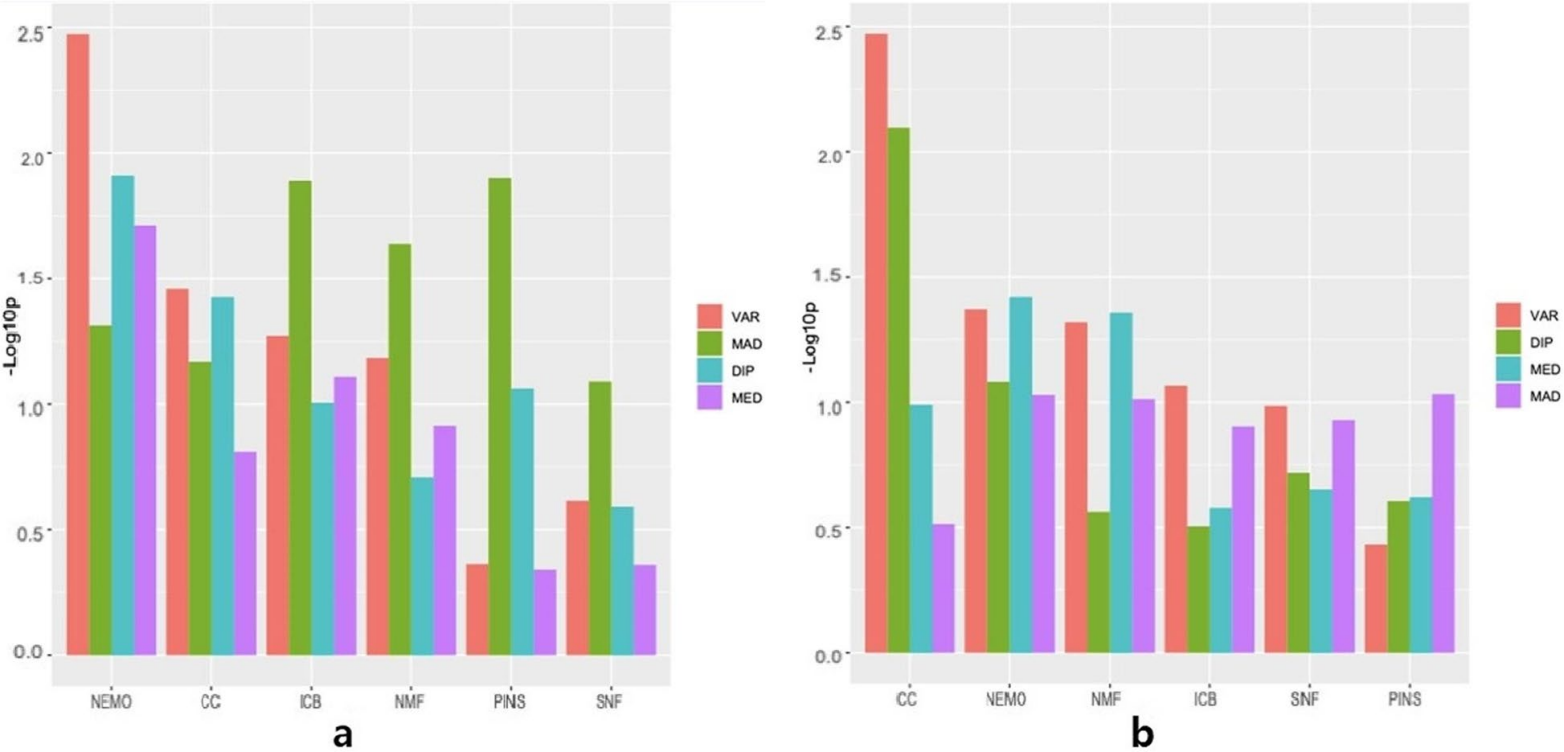
Average of -log_10_(*p*-value) in the log-rank test for four cancer datasets using **a** 500 genes and **b** 2000 genes

Figure 2 shows the average log-rank *p*-values of the four datasets for each combination of methods. This criterion shows whether the clustered subtypes actually showed significant differences in the survival profile. When using 500 selected genes, as shown in Fig. 2(a), VAR showed the best performance (i.e., the smallest *p*-value) when used in combination with NEMO and CC, while DIP showed the second-best performance in combination with NEMO and CC. However, MAD outperformed the other feature selection methods when used in combination with all other clustering methods except for NEMO and CC. When using 2000 selected genes as shown in Fig. 2(b), the variance method consistently showed decent performance in combination with all clustering methods except for PINS. Focusing on the clustering methods, NEMO showed the best performance overall in 500 selected genes and the second-best performance in 2000 selected genes, after CC. The details for the log-rank test *p*-values for each dataset can be found in Tables 4 and Table 5—the former table for 500 features and the latter for 2000 features. Although no single combination was found to have a distinctively good performance, CC and NEMO, as previously mentioned, had a tendency to show lower *p*-values in general. We also noted that NEMO again showed a tendency to produce a large number of clusters when feature selection was done prior to clustering, as in the case when feature selection was not done a priori.

**Table 4:** Log-rank test *p*-values for combinations of methods with 500 features

FS	Dataset	Subtyping Identification Methods					
		CC	PINS	NMF	ICB	SNF	NEMO
MED	AML	0.42 (4)	0.38 (3)	0.13 (3)	0.12 (4)	0.17 (2)	2.0E-03 (7)
	GBM	0.01 (4)	0.40 (3)	0.02 (3)	0.01 (4)	0.83 (2)	6.6E-04 (6)
	BIC	0.35 (4)	0.37 (2)	0.45 (2)	0.17 (5)	0.30 (2)	0.35 (6)
	COAD	0.39 (6)	0.77 (2)	0.19 (3)	0.18 (5)	0.86 (2)	0.31 (8)
VAR	AML	9.0E-04 (3)	0.10 (5)	0.06 (5)	3.0E-03 (4)	0.18 (2)	0.01 (5)
	GBM	0.09 (3)	0.73 (2)	0.09 (2)	0.06 (5)	0.73 (2)	7.0E-07 (6)
	BIC	0.04 (4)	0.52 (2)	0.53 (2)	0.14 (5)	0.06 (4)	0.09 (4)
	COAD	0.05 (4)	0.96 (2)	0.01 (2)	0.32 (4)	0.44 (2)	0.29 (8)
MAD	AML	1.0E-03 (4)	0.05 (2)	0.04 (4)	1.0E-04 (4)	0.21 (2)	0.08 (6)
	GBM	0.09 (3)	2.2E-05 (5)	0.05 (2)	0.05 (5)	0.04 (3)	1.0E-03 (6)
	BIC	0.32 (6)	0.24 (3)	0.06 (2)	0.09 (4)	0.06 (4)	0.12 (4)
	COAD	0.55 (2)	0.09 (3)	2.0E-03 (3)	0.06 (5)	0.10 (3)	0.46 (9)
DIP	AML	0.01 (6)	0.75 (2)	0.66 (2)	0.39 (4)	0.62 (2)	0.58 (2)
	GBM	0.01 (4)	0.02 (6)	0.23 (5)	0.02 (5)	0.20 (2)	0.08 (9)
	BIC	0.06 (2)	0.92 (2)	0.49 (2)	0.68 (4)	0.74 (2)	0.48 (3)
	COAD	0.27 (2)	4.0E-03 (2)	0.02 (2)	0.02 (3)	0.05 (2)	0.00 (5)

Value in parentheses indicates the number of clusters $k$

**Table 5:** Log-rank test *p*-values for combinations of methods with 2000 features

FS	Dataset	Subtyping Identification Methods					
		CC	PINS	NMF	ICB	SNF	NEMO
MED	AML	0.29 (2)	0.01 (6)	0.04 (4)	0.27 (6)	0.03 (6)	5.0E-03 (5)
	GBM	2.0E-03 (3)	0.66 (4)	0.05 (5)	0.26 (4)	0.74 (6)	0.01 (6)
	BIC	0.44 (3)	0.64 (2)	0.04 (3)	0.08 (4)	0.24 (5)	0.51 (5)
	COAD	0.43 (4)	0.56 (2)	0.05 (3)	0.83 (5)	0.56 (3)	0.10 (8)
VAR	AML	3.4E-07 (6)	0.07 (2)	0.06 (2)	0.06 (6)	0.10 (2)	0.03 (3)
	GBM	0.10 (3)	0.75 (2)	0.02 (2)	0.09 (4)	0.05 (3)	2.0E-03 (7)
	BIC	0.02 (3)	0.64 (2)	0.20 (2)	0.10 (4)	0.11 (4)	0.42 (6)
	COAD	0.23 (3)	0.58 (2)	0.02 (4)	0.11 (5)	0.20 (3)	0.15 (9)
MAD	AML	0.67 (3)	0.01 (5)	0.11 (5)	0.10 (5)	0.23 (2)	0.05 (7)
	GBM	0.24 (4)	0.03 (2)	0.02 (2)	0.03 (4)	3.6E-03 (4)	0.04 (6)
	BIC	0.21 (3)	0.32 (3)	0.32 (2)	0.16 (4)	0.40 (5)	0.10 (7)
	COAD	0.26 (4)	0.96 (2)	0.11 (4)	0.54 (5)	0.59 (3)	0.42 (3)
DIP	AML	7.0E-04 (5)	0.92 (2)	0.80 (2)	0.69 (4)	0.58 (2)	0.01 (5)
	GBM	1.3E-04 (3)	0.68 (3)	0.42 (3)	0.14 (4)	0.28 (2)	0.04 (6)
	BIC	0.13 (3)	0.78 (2)	0.48 (2)	0.33 (4)	0.36 (2)	0.15 (3)
	COAD	0.35 (4)	0.01 (2)	0.04 (2)	0.30 (3)	0.02 (2)	0.51 (4)

Value in parentheses indicates the number of clusters $k$

### Silhouette score

**Fig. 3:**
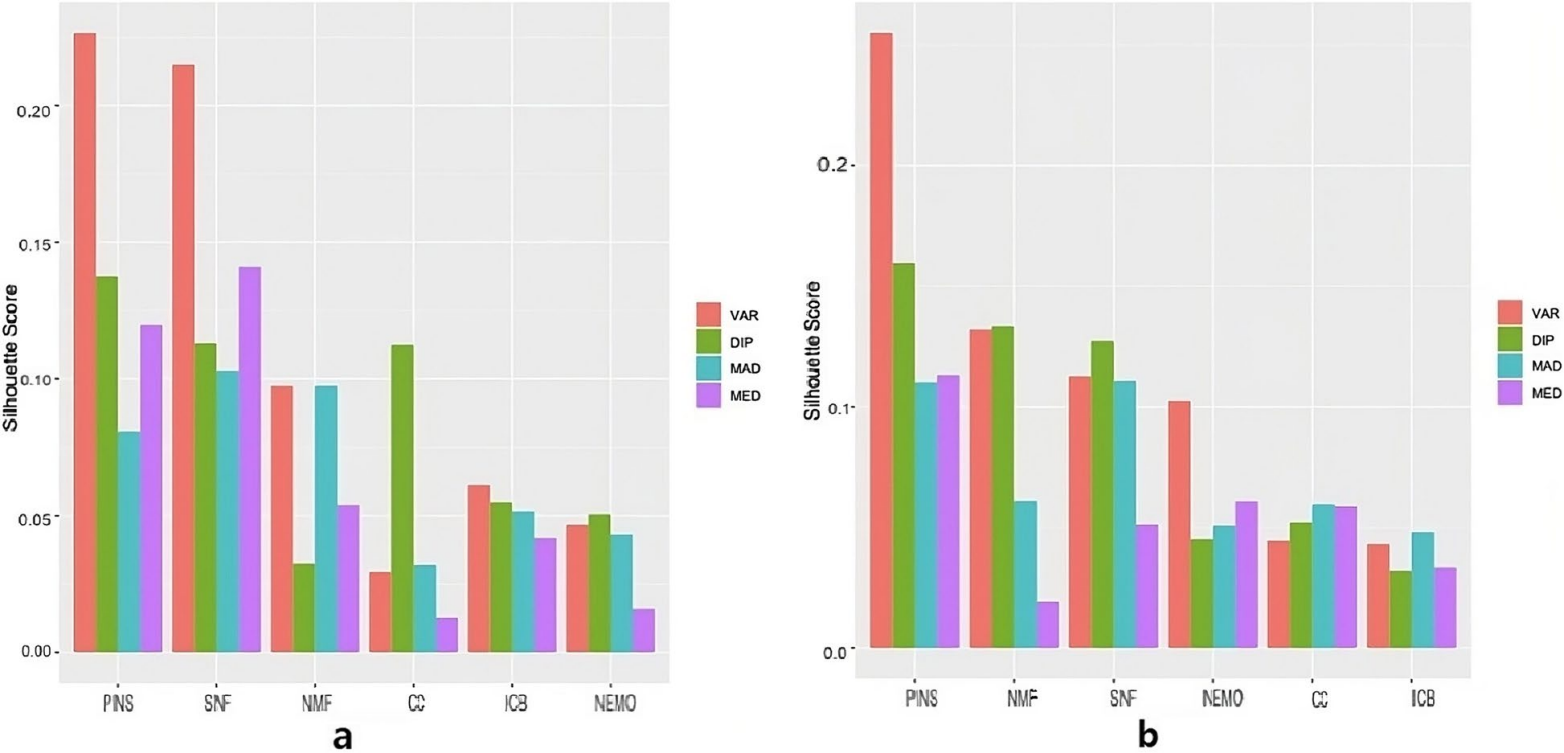
Average silhouette scores for four cancer datasets using **a** 500 genes and **b** 2000 genes

Figure 3 shows the average silhouette scores of the four cancer datasets. All silhouette scores with negative values were changed to zero. According to the silhouette score, VAR generally showed a decent performance relative to the other feature selection methods and PINS relative to the other clustering methods. The combinations of variance with PINS and SNF similarly performed well when 500 genes were used for clustering, whereas the combination of variance with PINS only showed adequate performance when 2000 genes were used. Overall, the results for the log-rank test and silhouette scores revealed that VAR can be recommended as a feature selection method, whereas the performance differed considerably among the subtype identification methods.

### Accuracy

Tables 6 and 7 show accuracy in terms of ARI and NMI. For the BIC datasets, NMF showed the best performance in terms of ARI in combination with all unsupervised feature selection methods, while it showed the worst performance without feature selection (Table 6). It showed especially good performance in combination with MCFS or VAR. For the combination with supervised feature selection such as mRMR or MCFS, NMF showed the second best performance after PINS. In terms of NMI, SNF was found to be highly vulnerable to the feature selection method, and PINS showed poor performance overall, especially when used with MED. Other than those two methods, the remaining subtyping methods had similar NMI values. ICB had decent performance when used alone, especially in the BIC dataset, where it showed the best ARI and NMI among the clustering algorithms without feature selection. However, both measures always either remained similar or worsened in combination with feature selection methods. The supervised feature selection methods, mRMR and MCFS, showed overall high accuracy as expected, except when CC or ICB was used for subtype identification when 2000 features were selected.

**Table 6:** Adjusted Rand Index (ARI) and Normalized Mutual Information (NMI) for the BIC dataset

FS			Subtyping Identification Methods					
			CC	PINS	NMF	ICB	SNF	NEMO
w/o FS		ARI	0.26	0.26	0.01	0.29	0.26	0.26
		NMI	0.30	0.30	8.5E-04	0.39	0.36	0.35
MED	500	ARI	0.19	0.07	0.31	0.19	0.08	0.17
		NMI	0.27	0.05	0.24	0.27	0.05	0.25
	2000	ARI	0.23	0.05	0.26	0.25	0.22	0.19
		NMI	0.27	0.02	0.30	0.34	0.31	0.27
VAR	500	ARI	0.23	0.32	0.40	0.25	0.26	0.26
		NMI	0.32	0.23	0.30	0.34	0.37	0.33
	2000	ARI	0.30	0.32	0.45	0.30	0.26	0.21
		NMI	0.33	0.23	0.34	0.38	0.37	0.32
MAD	500	ARI	0.24	0.26	0.40	0.26	0.24	0.24
		NMI	0.32	0.32	0.29	0.33	0.34	0.33
	2000	ARI	0.27	0.26	0.37	0.26	0.22	0.20
		NMI	0.31	0.30	0.26	0.33	0.31	0.29
DIP	500	ARI	0.37	0.33	0.38	0.24	0.18	0.27
		NMI	0.30	0.24	0.33	0.33	0.16	0.35
	2000	ARI	0.27	0.37	0.39	0.23	0.24	0.28
		NMI	0.33	0.31	0.33	0.35	0.21	0.37
mRMR	500	ARI	0.29	0.42	0.41	0.27	0.29	0.28
		NMI	0.32	0.32	0.28	0.34	0.36	0.36
	2000	ARI	0.26	0.43	0.42	0.23	0.30	0.27
		NMI	0.30	0.31	0.29	0.31	0.37	0.35
MCFS	500	ARI	0.28	0.45	0.41	0.22	0.30	0.30
		NMI	0.32	0.34	0.28	0.31	0.37	0.36
	2000	ARI	0.004	0.43	0.41	0.003	0.29	0.24
		NMI	0.01	0.31	0.27	0.01	0.36	0.33

**Table 7:** Adjusted Rand Index (ARI) and Normalized Mutual Information (NMI) for the COAD dataset

**FS**			**Subtyping Identification Methods**					
			CC	PINS	NMF	ICB	SNF	NEMO
**w/o FS**		ARI	0.23	0.23	0.00	0.19	0.23	0.23
		NMI	0.20	0.21	0.00	0.19	0.23	0.20
MED	500	ARI	0.06	0.00	0.09	0.12	0.01	0.09
		NMI	0.12	4.3E-03	0.11	0.14	0.01	0.12
	2000	ARI	0.11	0.00	0.13	0.13	0.24	0.10
		NMI	0.17	2.7E-03	0.14	0.16	0.20	0.14
VAR	500	ARI	0.15	0.00	0.11	0.11	0.00	0.09
		NMI	0.13	3.0E-03	0.07	0.12	2.2E-03	0.16
	2000	ARI	0.20	0.07	0.27	0.12	0.21	0.12
		NMI	0.19	0.05	0.23	0.15	0.21	0.16
MAD	500	ARI	0.02	0.14	0.15	0.19	0.17	0.09
		NMI	0.04	0.15	0.12	0.17	0.18	0.14
	2000	ARI	0.13	1.3E-03	0.19	0.17	0.25	0.25
		NMI	0.15	0.02	0.20	0.17	0.24	0.25
DIP	500	ARI	0.00	0.02	0.00	0.00	3.4E-03	0.10
		NMI	0.01	0.01	0.01	0.03	4.3E-03	0.12
	2000	ARI	0.19	0.03	0.02	2.8E-03	1.9E-04	0.17
		NMI	0.19	2.3E-03	0.01	0.01	0.01	0.15
mRMR	500	ARI	0.16	0.26	0.23	0.27	0.28	0.27
		NMI	0.14	0.17	0.09	0.34	0.24	0.24
	2000	ARI	0.18	0.22	0.06	0.13	0.27	0.22
		NMI	0.16	0.20	0.01	0.14	0.23	0.21
MCFS	500	ARI	0.18	0.23	0.32	0.07	0.21	0.18
		NMI	0.18	0.20	0.25	0.13	0.19	0.18
	2000	ARI	0.01	0.12	0.28	0.00	0.25	0.21
		NMI	0.03	0.14	0.22	0.01	0.21	0.20

In the case of the COAD dataset, values of ARI and NMI were small in all methods (Table 7). However, as in the BIC data, the performance of NMF without feature selection was very low, and the performance of supervised feature selection mRMR and MCFS was good. In addition, there were many cases where the performance of NMF and SNF was the best. In unsupervised feature selection methods, the values of ARI and NMI increased when the number of selected features was large, but in mRMR and MCFS, the accuracy tended to decrease when the number of selected variables was 2000 rather than 500. This seems to be because in the two supervised feature selection methods, selecting a large number of variables results in more redundant variables being selected.

**Fig. 4:**
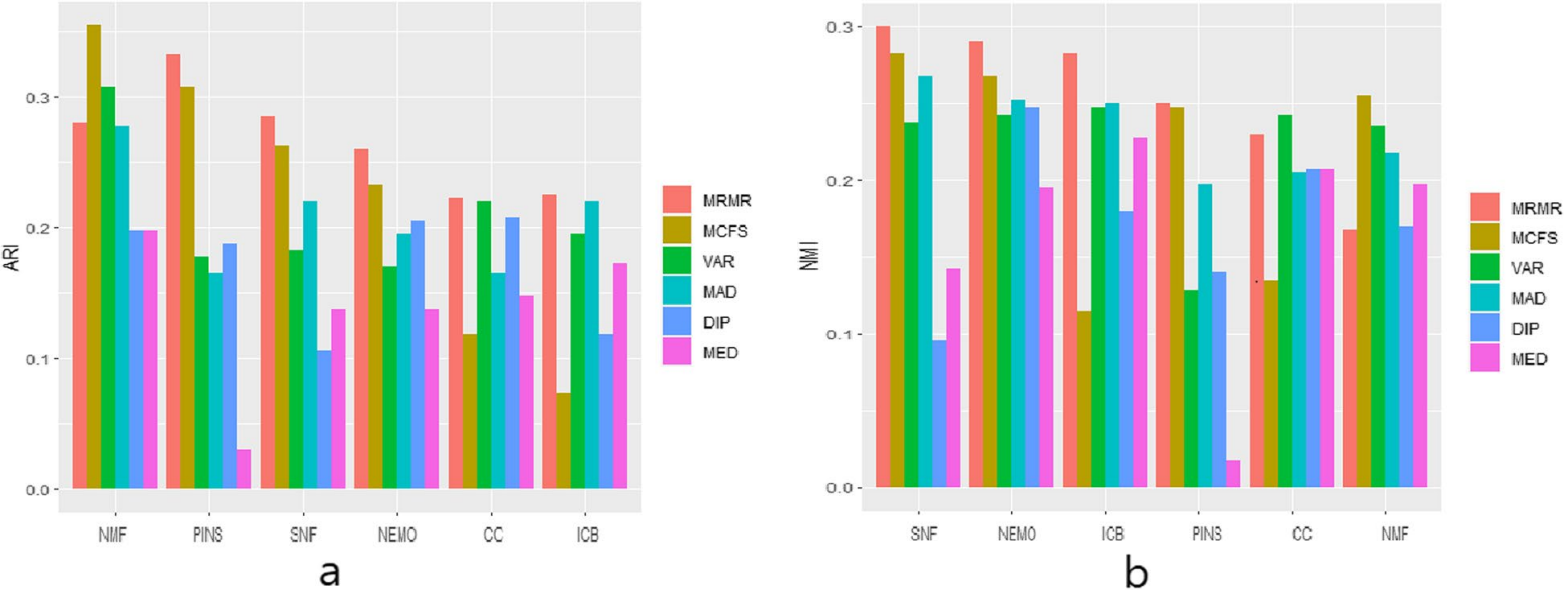
Average accuracy for four cancer datasets, **a** Adjusted Rand Index (ARI) and **b** Normalized Mutual Information (NMI)

Figure 4 shows the average ARI and NMI values for the BIC and COAD datasets when 500 and 2000 genes are selected. It can be seen that the result for ARI is consistent with the conclusions that can be drawn from Tables 6 and 7.

### Time complexity

Table 8 shows the time complexity of six subtyping identification methods without feature selection for four datasets and six feature selection methods for two datasets. The running time was obtained using Ubuntu on an Intel i9 processor with 64 GB of memory. Among the subtyping identification methods, NMF showed an overwhelmingly long running time, and ICB showed the second longest running time. On the contrary, SNF and NEMO took the shortest time to run for all datasets. Among the feature selection methods, the running time of MCFS, which is based on the Monte-Carlo approach, showed a running time that far exceeded other methods, as expected. DIP had the second longest running time, but there was a major difference from that of MCFS. mRMR, showed a fairly fast execution time despite using the greedy search algorithm. While mRMR showed different times depending on the dataset, VAR showed a stable and short running time regardless of the dataset or number of selected features.

**Table 8:** Time complexity for feature selection methods and subtyping methods

**Subtyping Identification Methods without FS**							
		CC	PINS	NMF	ICB	SNF	NEMO
AML		5.18	20.64	1130.33	870.40	2.19	2.18
GBM		37.29	131.21	1982.45	870.40	6.00	5.87
BIC		53.33	95.90	7573.64	1334.64	9.76	9.25
COAD		8.83	33.84	1809.96	1058.59	1.41	1.36
**Feature Selection Methods**							
		MED	VAR	MAD	DIP	mRMR	MCFS
COAD	500	0.87	0.78	1.15	7.43	0.53	30,078.44
	2000	0.86	0.18	1.15	5.49	2.05	30,078.44
BRCA	500	2.71	0.90	3.10	8.12	1.53	108,000.00
	2000	1.06	1.53	2.40	8.64	3.72	108,000.00

Numbers indicate running time (unit: second)

Since subtype identification is performed after variable selection, the time required for each combination is the sum of the respective times.

### Guideline

Based on the above results, guidelines for the appropriate choice of a variable selection method and subtype identification method are summarized as follows. First, if there is sufficient information on the relevant phenotype and the dataset is not very large, mRMR or MCFS is good as a variable selection method in terms of the accuracy criterion. In this case, PINS, NMF or SNF are also good choices for subtype identification. Otherwise, when unsupervised feature selection is used due to insufficient phenotypic information, NMF and SNF are still good choices as subtype identification methods. In this case, it is recommended not to use NMF alone without variable selection, and not to use SNF together with the DIP and MED methods. Second, in terms of the significance test for survival times among groups, small *p*-values were shown when CC or NEMO was used with VAR. However, when the number of selected genes was sufficiently large, NMF showed stable, small *p*-values unless DIP was used for feature selection. Third, in terms of computation time, MCFS does not seem to be suitable for large datasets. All other feature selection methods have very short running times, and there is no difference in time except for DIP. Among the subtype identification methods, NMF and ICB took much more time than others.

In summary, as a feature selection method, we recommend VAR, which shows good performance for most subtype identifications. For subtype identification, we recommend NMF, which stably shows good performance in many cases.

## Discussion

We compared six subtyping methods, two of which are used only for single-omics data sets, and four for both single-omics and multi-omics data sets. This study is different from previous comparative research in that it compared the performance of cancer subtyping methods combined with various feature selection methods. Rather than a single method dominating the others, the best methodology depended on the data used, the number of features selected, and the evaluation method.

This result is supported by the study of Dhal and Azad [24], which showed that the performance of the feature selection methods varied significantly across different data types. In a comparative study of subtype identification methods, different methods were selected as the best for each task. For example, multiple canonical correlation analysis was selected as the best for multi-omics data among seven subtyping methods in terms of *p*-values for the log-rank test in differential survival [59], while the regularized multiple kernel learning algorithm showed the best performance for single omics data [60]. PINS [18] and CIM showed the largest number of significant *p*-values in another study [43]. However, moCluster showed the best performance in a simulation study [58]. The authors compared the ability of the methods to classify the samples into the correct subgroups, rather than using *p*-values of the log-rank test [38]. ICluster and moCluster showed better performance than other methods [39]. Sensitivity and the ability to recover the number of clusters and common specific structures across datasets were considered as evaluation criteria. Since each evaluation criterion has its own advantages and disadvantages, no single criterion can be considered as the gold standard. It has been reported that the silhouette value decreases and approaches zero as the number of dimensions increases [18]. Silhouette values do not necessarily indicate a clinical association itself, and its usefulness is limited for high-dimensional data due to noise [18]. Meanwhile, the *p*-value for the log-rank test may not fully represent the clustering ability of the algorithms, since some patient groups may have a similar survival distribution even though they fall into different cancer subtypes or vice versa.

The number of clusters chosen for each clustering method was not set to be the same for all methods, as being able to evaluate the optimal number of clusters is also considered as an aspect of the algorithm’s performance. Except for CC and ICB, which require the user to evaluate the optimal number of clusters subjectively, all methods used their inherent algorithms to assess the number of clusters, with the maximum number of clusters set as 10 for all algorithms.

Although the results of all four datasets tell us that there is no single combination of methods that outperforms others, the CC and NMF methods were generally good choices among the six clustering methods when informative genes were selected prior to clustering in terms of differences in the survival curves. Especially for CC, the combination of CC with variance as a feature selection method showed the best performance in terms of *p*-values of the log-rank test in the two datasets of AML and BIC, and the combination of CC with DIP showed the best performance in the GBM dataset.

The variance method and DIP were often included in the best combinations. In the COAD dataset, the combination of DIP and the PINS clustering method showed the best performance in terms of *p*-values of the log-rank test. The drawback of CC is that the number of clusters k is subject to the user’s opinion, and that its performance is sensitive to the value of k. Therefore, it is recommended to carefully set k using additional measures such as the silhouette score. Overall, the results of selecting 500 and 2000 genes differed by combinations and datasets.

We acknowledge that our study has some limitations. Firstly, we only utilized filter methods for feature selection; these methods have the advantage of reducing computational time and being efficient for high-dimensional datasets, but they may overlook certain relevant features. Secondly, our analysis employed only a subset of TCGA data (namely, single-omics datasets), limiting the generalizability of our findings. Future research could explore the use of embedded feature selection methods or incorporate multi-omics data. In our opinion, it is also crucial to investigate more novel and recent feature selection methods to further enhance the performance of subtype identification in gene selection.

## Data Availability

The real data used to support the findings of this study are available from The Cancer Genome Atlas (TCGA) at http://cancergenome.nih.gov/tcga.
